# Periradicular Surgery of Human Permanent Teeth with Calcium-Enriched Mixture Cement

**Published:** 2013-08-01

**Authors:** Saeed Asgary, Sara Ehsani

**Affiliations:** a Iranian Center for Endodontic Research, Research Institute of Dental Sciences, Shahid Beheshti University of Medical Sciences, Tehran, Iran; b Dental Research Center, Research Institute of Dental Sciences, Shahid Beheshti University of Medical Sciences, Evin, Tehran, Iran

**Keywords:** Apicoectomy, Calcium-Enriched Mixture, Cementogenesis, Dental Cements, Endodontics, Oral Surgery, Periodontal Ligament, Root-end Filling Materials

## Abstract

**Introduction:**

Root-end preparation and restoration with an endodontic material are required when nonsurgical endodontic retreatment has failed or is impossible. The present clinical study reports the treatment outcomes of periradicular surgery using calcium-enriched mixture (CEM) cement.

**Materials and Methods:**

A prospective outcome study of periradicular surgery using CEM was conducted on 14 permanent teeth with persistent apical periodontitis. Using a standardized surgical protocol, 2-3 mm of the root apex was resected; approximately 3 mm deep root-end cavities were ultrasonically prepared and filled with CEM cement. All patients were available for recall.

**Results:**

Clinical and radiographic examination revealed complete healing of periradicular lesions, i.e. regeneration of periodontal ligament and lamina dura in 13 teeth (93% success) during a mean time of 18 months; moreover, the teeth were functional and asymptomatic.

**Conclusion:**

Favorable treatment outcomes in this prospective clinical study suggested that CEM cement may be a suitable root-end filling biomaterial.

## Introduction

When conventional root canal treatment (RCT) has failed, non-surgical retreatment is the preferred option in the mainstream of cases. In a number of cases, several factors such as a complex root canal system or previous procedural accidents may impede the success of nonsurgical retreatment. In such cases, periradicular surgery would be the treatment of choice in order to save the tooth [1].

After root-end resection and preparation, the root canal filling material is placed within the created cavity to close the path of communication between infected root canal system and periradicular tissues. Using a root canal filling material with ideal properties will have an immense effect on the treatment outcomes of the surgery. An ideal root canal filling material should be non-absorbable, non-corrosive, non-cytotoxic, not affected by moisture, dimensionally stable, biocompatible, antibacterial, radiopaque, cost-effective, easily manipulated, adhesive to the dentinal walls, and able to create a tight seal as well as to induce cementogenesis [2-4]. Numerous materials have been recommended for root-end fillings and many studies have attempted to identify an ideal one; however an ideal material has not yet been found [5].

Mineral trioxide aggregate (MTA) was introduced to create an effective seal between the root canal system and the periradicular tissues [6, 7]. As a root canal filling material, MTA has proved successful [8, 9]; comparative studies with other root canal filling materials have shown less leakage as well as excellent periradicular healing when used as a root-end filling material or apical plug [10]. Despite its outstanding tissue compatibility [11] and great impact in endodontic practice [12], MTA has some shortcomings including questionable antimicrobial activity, delayed setting time, decreased flexural strength, poor handling characteristics and high cost price [13, 14].

Calcium-enriched mixture (CEM) cement was also introduced as root-end filling biomaterial. It proved to have coronal as well as retro-sealing ability equal to MTA [4, 15, 16] and acts as an inductive agent for dentinogenesis [17, 18], cementogenesis [19, 20] and osteogenesis [21]. CEM cement can set in aqueous environments and has suitable film thickness and flow [22]. Furthermore, its antibacterial effect is comparable to calcium hydroxide (CH) [23]; CEM also reduced the neuronal activity similar to MTA [24]. One unique aspect of CEM is its ability to form hydroxyapatite over resected roots and material surface even in normal saline; this demonstrates good biocompatibility [25].

The purpose of this prospective clinical study is to describe the clinical and radiographic outcomes of periradicular surgery in human permanent teeth using CEM cement as a root-end filling biomaterial.

## Material and Methods

Patients from both genders were required to have i) a symptomatic permanent tooth; ii) an acceptable root canal filling; and iii) a periradicular lesion of endodontic origin. Patients with moderate or severe marginal periodontitis, active systemic disease, physical or mental disability, or those who were pregnant or nursing were excluded. In cases with inadequate coronal restoration, a new coronal restoration was provided before surgical treatment. All recruited subjects were informed of the possible complications. Written informed consent was signed by all patients. Twelve patients (overall 14 teeth) were treated in an endodontic clinic (Table 1). Medical and dental histories of patients were recorded.

**Table 1.** Summary of pathology results, size of lesion, and outcomes of cases

Case No.	Patient No. (Gender)	Patient Age (Years)	Tooth No. (FDI)	Size of Lesion	Pathology/Diagnosis	Follow-up (months)	Outcome
**1**	1 (M)	42	16	Small	Granuloma	15	Healed
**2**	2 (F)	25	36	Large	Granuloma	22	Healed
**3**	3 (M)	34	31	Large	Cyst	19	Healed
**4**	4 (M)	23	28	Small	Granuloma	14	Healed
**5**	5 (F)	37	11	Moderate	Granuloma	26	Healed
**6**	5 (F)	37	21	Moderate	Granuloma	26	Healed
**7**	6 (M)	52	21	Small	Cyst	24	Healed
**8**	7 (F)	29	12	Large	Cyst	13	Healed
**9**	8 (M)	46	22	Large	Granuloma	12	Healed
**10**	8 (M)	29	25	Moderate	Granuloma	15	Healed
**11**	9 (F)	25	46	Large	Cyst	13	Failed
**12**	10 (F)	38	26	Small	Granuloma	24	Healed
**13**	11 (F)	57	12	Moderate	Cyst	17	Healed
**14**	12 (F)	44	27	Small	Granuloma	12	Healed

In the operative session, a 400 mg tablet of Ibuprofen was prescribed to prevent post-treatment pain and discomfort [26]. An antiseptic mouthwash (0.2% chlorhexidine gluconate) was provided and teeth were locally anesthetized with 2% Lidocaine containing 1:80000 adrenaline (DarouPakhsh, Tehran, Iran).

A sulcular incision followed by a full thickness mucoperiosteal buccal flap provided adequate access to the region; a Luebkhe-Ochsenbein flap design was preferred for incisors for aesthetic reasons [27]. Buccal bone osteotomy was completed using a slow-speed handpiece accompanying with copious amounts of sterile normal saline. Periradicular lesion was removed and sent for histopathologic examination. Approximately 3-mm root-end resection was performed perpendicular to the long axis of the root. A 3-mm deep root-end cavity was prepared ultrasonically, powered by a minipiezon with DT-043 ultrasonic retrotip (EMS, Nyon, Switzerland). CEM cement (BioniqueDent, Tehran, Iran) powder and liquid was mixed according to manufacturer’s instructions and placed into the root-end cavity.

All radiographs were taken with Suni Ray digital charge-coupled device (CCD) intraoral sensor (Suni Medical Imaging Inc., CA, USA) with bisecting method and evaluated in a room with a dimmed light; after radiographic confirmation of proper placement of the biomaterial, the reflected tissues were repositioned, sutured with polyvinylidene fluoride (PVDF; CG, Tehran, Iran) [28] and compressed with moist gauze for 3 min. All patients were given verbal and written postsurgical instructions. They were seen 5 days postoperatively for clinical evaluation and suture removal.

Clinical and radiographic criteria according to the “Quality Guidelines” of European Society of Endodontology (ESE) for favorable outcomes were as follows: absence of pain, swelling and other symptoms, satisfactory healing of soft tissue, no sinus tract, no loss of function and radiological evidence of repair of apical periodontitis including reformation of the periodontal ligament space [29]. Clinical and radiographic evaluations were performed 1+ year/s post-operatively. The lesions were considered small if they were smaller than 5 mm; moderate when 5-10 mm; and large when exceeded 10 mm.

## Results

A total of 14 mandibular or maxillary teeth in twelve patients were treated (Table 1). The average age of patients was 38 years (23-57 year). All patients (5 males and 7 females) were available for follow-up. All cases had marginal bone height of at least 3 mm. The initial size of the lesions can be seen in Table 1.

**Figure 1.**
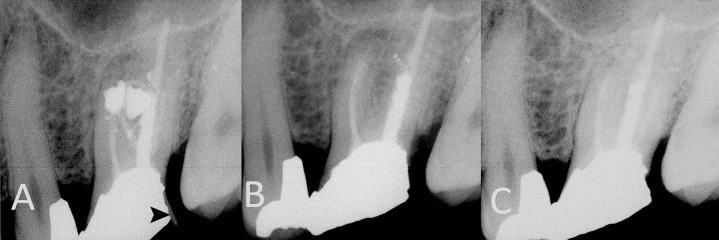
A) Periapical radiograph of a maxillary molar with radiographic masses of amalgam, the black arrowhead shows the path of gutta-percha used for finding the course of sinus tract (case No. 12); B) Immediately after endodontic surgery; C) the tooth 2 years after surgery

**Figure 2.**
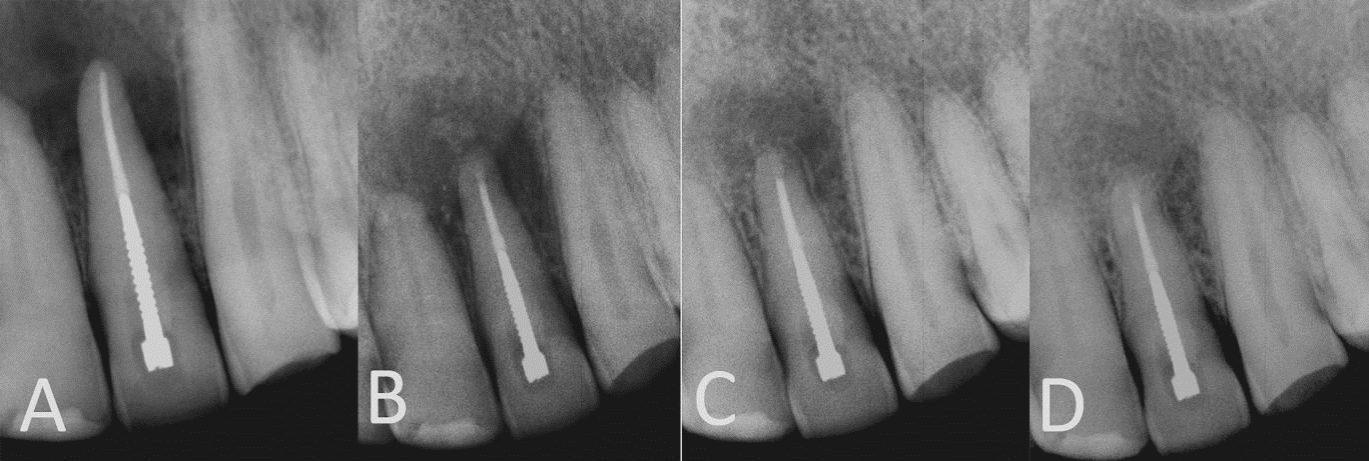
A) A maxillary left lateral incisor with extensive periradicular lesion (case No. 9); B) immediately after periradicular surgery; C) 4 months after surgery; D) one-year follow-up radiograph shows normal PDL

**Figure 3.**
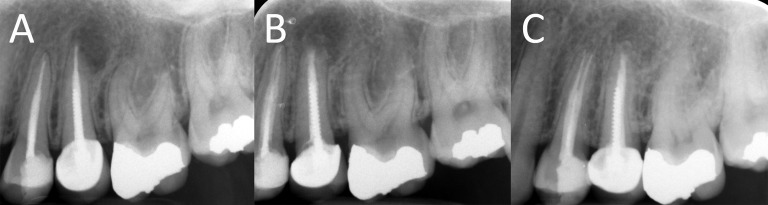
A) A maxillary premolar with moderate apical lesion (case No. 10); B) immediate postoperative radiograph; C) the tooth 15 months after root-end surgery shows complete periradicular healing

The mean time for recall was 18 months (12-26 months). There was no clinical sign of inflammation and/or infection except in one case (success equal to 93%). Cases 4 and 14 were maxillary left second and third molar, whose surgical approach was difficult; treatment outcomes were, however, satisfactory and the lesions successfully healed. The case illustrated in Figure 1 was the only re-operation case, which came to see us with a symptomatic periradicular lesion, persisting after periradicular surgery. Favorable outcomes were achieved according to the “Quality Guidelines” of ESE. Radiographic examination revealed normal periodontium in all healed teeth (Figures 1, 2 and 3).

## Discussion

Although non-surgical retreatment is successful in most case of endodontic failure, there are cases in which periradicular surgery is necessary to save the tooth. The success of periradicular surgery is, in part, dependent on the selected root canal filling material. In a recent systematic review, investigators found a success rate of 77.8% for periradicular surgery at 2-4 years and a rate of 71.8% at 4-6 years [30]. Another high level of evidence report revealed that out of 2,788 endodontically-treated, 330 teeth required re-surgery and only 35.7% of them had healed after 1 year [31]. In the present study, CEM cement was applied in periradicular surgery and proved to be successful (93%) as a root-end filling biomaterial.

The high clinical success rate may be the result of a number of physical and biochemical characteristics. A crucial characteristic for an ideal root canal filling material is the ability to stimulate periradicular tissue regeneration, in particular, cementogenesis over the material [8, 9]. Previous studies on CEM cement revealed that this material is capable of inducing hard tissue formation, in particular, cementogenesis [19, 20]. In a recent study, histological evaluation demonstrated that CEM cement and MTA have similar favorable biological effects in furcation perforation repair cases, especially in inducing the formation of cementum-like hard tissue bridges, which was observed in all specimens [20]. A possible reason is that mixed CEM cement produces a considerable amount of hydroxyl, calcium, and phosphate ions in the presence of water, which results in increased pH and the formation of hydroxyapatite crystals, a naturally-made material in hard tissues; a recent scanning electron microscopy (SEM) and energy dispersive X-ray analysis (EDXA) demonstrated that surface topography was altered by hydroxyapatite crystal formation on CEM root-end fillings in all samples [25]. Moreover, the composition and structure of precipitated crystals were comparable with that of standard hydroxyapatite. The fact that CEM cement can make hydroxyapatite over its surface, even in normal saline solution, means that the process is independent of exogenous sources. It should be mentioned that other biomaterials do not show this phenomenon in normal saline solution.

A randomized controlled animal study demonstrated that both CEM cement and MTA induced periradicular tissue healing regeneration including the production of cementum and new bone, when used as root-end filling biomaterials [19]. In an ideal situation, a root-end filling should induce the regeneration of periodontal ligament and cementum in addition to new bone formation [32]. CEM cement has the ability to promote cementogenesis over both the root-end dentinal surface and the material. A remarkable feature was that the newly formed eosinophilic cementum contained entrapped cementocytes and periodontal ligament fibers insertions [19]. Uninterrupted cementum coverage over the root-end filling and surrounding dentin is a significant quality; it can serve as a barrier against the destructive residual content within the root canal system. Another quality that may be effective in tissue regeneration is the similarity of the distribution pattern of calcium, phosphorus, and oxygen on the surface of CEM cement and surrounding dentin [33]. The exact biochemical mechanism of cementogenesis by CEM cement has not been discovered yet.

The egress of bacteria and/or their byproducts into the periradicular tissues results in tissue inflammation. Root canal filling materials are meant to eradicate any remnant bacteria and to seal communications between the root canal and tooth and its external environment [6]. Consequently, successful periradicular surgery depends to a great extent on the achievement of an ideal apical seal. Insufficient apical seal has been suggested to be the major cause of endodontic surgical failure. A previous study demonstrated that the sealing ability of CEM cement and MTA is equivalent, and they are both significantly better than Intermediate Restorative Material (IRM) [15]; in terms of coronal sealing in endodontically treated teeth, CEM and MTA are also more effective than amalgam and composite resin [16]. In addition, shorter setting time, more flow, and considerably less film thickness were achieved with CEM when compared with MTA [22]. These physical properties considerably affect the material’s clinical performance. For instance, the slight expansion and reasonable flow and film thickness can ensure an effective seal after setting. Another critical factor influencing the rate of hydration and, accordingly, the strength and setting characteristics of cement is the fineness of its particles; among Root MTA, calcium hydroxide, and CEM cement, the smallest range of particle size belonged to CEM cement [34].

## Conclusion

The considerable success rate of this clinical study indicates that CEM cement may be considered an acceptable endodontic biomaterial for periradicular surgeries. Further randomized clinical trials with long-term follow-up and larger sample size are recommended.
